# Beneficial rhizosphere bacteria provides active assistance in resisting *Aphis gossypii*s in *Ageratina adenophora*

**DOI:** 10.3389/fpls.2024.1394153

**Published:** 2024-05-15

**Authors:** Youxin Yu, Zihao Yang, Mengyang Han, Shengnan Sun, Gang Xu, Guoqing Yang

**Affiliations:** ^1^ College of Plant Protection, Yangzhou University, Yangzhou, China; ^2^ College of Animal Science and Technology, Yangzhou University, Yangzhou, China

**Keywords:** *Ageratina adenophora*, *Bacillus cereus*, *Aphis gossypii*, soil physicochemical properties, native polyphagous insect

## Abstract

*Ageratina adenophora* can enhance its invasive ability by using beneficial rhizosphere bacteria. *Bacillus cereus* is able to promote plant growth and provide a positive feedback effect to *A. adenophora*. However, the interaction between *A. adenophora* and *B. cereus* under the influence of native polyphagous insect feeding is still unclear. In this study, *Eupatorium lindleyanum*, a local species closely related to *A. adenophora*, was used as a control, aimed to compare the content of *B. cereus* in the roots of *A. adenophora* and rhizosphere soil after different densities of *Aphis gossypii* feeding, and then investigated the variations in the population of *A. gossypii* and soil characteristics after the addition of *B. cereus*. The result showed that *B. cereus* content in the rhizosphere soil and root of *A. adenophora* increased significantly under *A. gossypii* feeding compared with local plants, which also led to the change of α-diversity and β-diversity of the bacterial community, as well as the increase in nitrate nitrogen (NO_3_^–^N) content. The addition of *B.cereus* in the soil could also inhibit the population growth of A. gossypii on *A. adenophora* and increase the content of ammonium nitrogen (NH_4_^+^-N) in the soil. Our research demonstrated that *B. cereus* enhances the ability of *A. adenophora* to resist natural enemy by increasing soil ammonium nitrogen (NH_4_^+^-N) and accumulating other beneficial bacteria, which means that rhizosphere microorganisms help invasive plants defend themselves against local natural enemies by regulating the soil environment.

## 1 Introduction

Plants and soil microbes collaborate to establish a favorable habitat for survival, which can have significant feedback on plant growth and fitness (Bever et al., 2012; Bowen et al., 2017). Rhizosphere microbial communities typically exhibit species-specificity (Singh et al., 2007). In the case of invasive alien plants, soil microorganisms can be harnessed to improve their ability to invade (Zhang et al., 2023). Various hypotheses of invasion mechanism have been proposed to explain this phenomenon, such as the enemy release hypothesis (Keane, 2002) and the mutualist hypothesis (Christian, 2001). Therefore, every element that modifies the interaction between plants and microbes would affect the capacity of nonnative plants to invade. For instance, Batten et al. (2006) discovered that two invasive species, *Centaurea solstitialis* and *Aegilops triuncialis*, seem to modify microbial communities in the areas they invade, which promotes their successful invasion. Furthermore, research has demonstrated that the invasive plant species, *Impatiens glandulifera*, can drastically alter soil microbial characteristics during only two growing seasons, leading to a notable increase in its invasiveness (Stefanowicz et al., 2019).

*Ageratina adenophora* (Sprengel), originally from Mexico in the Americas, was brought to Europe as a decorative plant and subsequently transferred to Australia and Asia (Darji et al., 2021). In the 1940s, it was introduced from Myanmar to the Chinese province of Yunnan (Wang and Wang, 2006). After 80 years of spreading and dispersing, *A. adenophora* has extensively colonized throughout south-west China (Du et al., 2022a). Research has also projected that the future range of *A. adenophora* in China will swiftly grow towards the east and north (Zhao et al., 2019). Moreover, *A. adenophora* has the ability to inhibit the growth of field crops and can establish a dominating population in the invaded region (Negi et al., 2023). Currently, research has summarized three potential mechanisms that promote the expansion of *A. adenophora*: phenotypic plasticity, allelopathy, and alteration of soil microbial community structure (Jiao et al., 2021; Khatri et al., 2022; Li et al., 2022).

*A. adenophora* faces several natural enemies as it spreads in natural habitats (Heystek et al., 2011). *Procecidochares utilis* Stone (Diptera: Tephritidae) is an obligatory parasitic insect that can induce galls on side branches of *A. Adenophora* (Gui et al., 2008), but its effectiveness as a biological control agent varies significantly across different regions (Sharma Poudel et al., 2020). For instance, its control effect is not satisfactory in the southwestern region of China (Yuan et al., 2021). Several studies have indicated that native predators can consume invasive plants within the introduced range (Niu et al., 2010; Van Driesche et al., 2010). According to Cheng et al. (2007), it was discovered that cotton *aphids* (*Aphis gossypii* Glover, Homoptera: Aphdiate) could complete its life cycle on *A. adenophora* as a native polyphagous insect, and *A. adenophora* growth rate was inhibited after *A.gossypii* feeding (Cheng et al., 2007). Furthermore, with the increase in *A.gossypii* density and feeding time, the content of secondary metabolites and the activity of antioxidant enzymes changed considerably (Lin et al., 2020).

*A. adenophora* has implemented many strategies to address external threats. Xia et al. (2023) discovered that the rhizosphere microbiota of *A. adenophora* harbors a population of plant growth-promoting rhizobacteria (PGPR) that could be utilized as bioinoculants to enhance the growth and productivity of these crops. For instance, invasive plants enhance their ability to invade by regulating soil microorganisms, such as *Bacillus*, which provide benefits to them, thereby inhibiting the growth of other native plants (Sun et al., 2013). The genus *Bacillus* can positively impact plant health through three mechanisms: (i) antagonistic activity against pests and pathogens, (ii) enhancement of host nutrition and growth, and (iii) stimulation of plant host defense (Choudhary and Johri, 2009). Such as *Bacillus cereus* strain D1 (BcD1) induces the production of several secondary metabolites and antioxidant enzymes in plants as a protective mechanism against heat stress and pathogen attack (Tsai et al., 2023). Zhang et al. (2020) identified six bacterial strains that were isolated from the roots of *A. adenophora*, including *B. cereus*. Previous research has demonstrated that *B. cereus* content increased with the invasion process of *A. adenophora* in the rhizosphere soil (Sun et al., 2021), and the growth of *A. adenophora* was extensively promoted after adding *B.cereus* (Du et al., 2023), due to the accumulation of *B. cereus* could alleviate its autotoxic allelopathy (Wu et al., 2023).

However, the precise contribution that *B. cereus* plays in facilitating the adaptation and defense against predators in *A. adenophora* remains uncertain. Consequently, this study aims to examine the response of beneficial bacteria in the rhizosphere of *A. adenophora* when exposed to *A. gossypii* feeding. Additionally, it intends to understand the adaptation mechanism of the *A. adenophora* under the interaction between rhizosphere microorganisms and predators.

## 2 Materials and methods

### 2.1 Plants and soil preparation

The seeds of *A. adenophora* were obtained from the Xishan District of Yunnan Province, and the Seeds of *E. lindleyanum* were purchased from Yunxiang Jingpin Flower Seedlings Store in Dali City, Yunnan Province. The seeds of both plants were germinated to the spouting stage and subsequently transplanted to a seedling tray (50 holes/tray). Once the plant reached a height of around 10 cm, it was moved to a plastic cup individually and placed in an artificial climate chamber with parameters set as follows: 25 ± 1°C, 60 ± 5% relative humidity, a photoperiod of 14 h light/10 h dark. The same nutrient soil was used for planting seedlings and transplanting soil, and the experiment was carried out in the greenhouse at Yangzhou University.

### 2.2 *B. cereus* preparation and insect rearing

*Bacillus cereus* ATCC14579 was used in the test (concentration maintained at 10^8^ CFU/ml), provided by the Institute of Plant Protection, Chinese Academy of Agricultural Sciences, and stored at a temperature of -20°C. The activated *B. cereus* was inoculated in 150 mL Luria Bertani (LB) medium and cultured at 28°C, 180 r/min for 24 hours to obtain the *B. cereus* culture broth.

The number of *B.cereus* was calculated by dilution broth plate colony counting method. After the serial dilution method was used to dilute the culture broth, the optical density (OD) value of each concentration of culture broth was measured, and 200 μL of culture medium was inoculated into LB ager medium. After 48 hours, the number of colonies in each culture dish was calculated to obtain the number of *B. cereus* (3 replicates at the same dilution). The calculation method: colony-forming unit (CFU) = the average number of three replicates of the same dilution × dilution multiple × 5, and the unit was CFU/mL.

*A.gossypii* was obtained from the natural host plant *Hibiscus syriacus* in the spring of 2021 and reared on *A. adenophora* and in the insect room (temperature (25 ± 1)°C, humidity (60 ± 5) %, photoperiod (14L:10D)).

### 2.3 Experimental design

#### 2.3.1 *A.gossypii* feeding on *A. adenophora*

*E. lindleyanum* was employed as a control to determine changes in *B. cereus* levels after *A. gossypii* feeding on *A. adenophora*. Seedlings of both plants were cultivated and then transplanted into plastic cups. 0, 5, 10, and 15 1st-instar *A.gossypii* nymphs were released on the top of the stem when the plants had grown to 10 cm in length. Until all the *A.gossypii* grew to the 4th-instar, all the plants were harvested, and the rhizosphere soil from the different treatments was carefully removed, which adhered to the root surface about 1 mm. All samples were sieved through a 40-mesh to determine the content of *B.cereus*, soil characteristics, and soil bacterial diversity in rhizosphere soil. In addition, plant roots from each treatment were cut for the detection of *B. cereus* content in five replicates.

#### 2.3.2 Adding *B. cereus* to the rhizosphere soil of *A. adenophora*

Seedlings of two plants were taken from plastic cups (same growth, plant height about 10 cm) in individual, and then 30 mL of sterile water (CK), 200-fold dilution of *B.cereus* (BC), and 200-fold dilution of Thiessen copper (TC, excluding bacterial treatment) suspension were added to the soil respectively. After that, 4th-instar nymphs with numbers of 0, 5, 10, and 15 were promptly released immediately after the treatment.

After 15 days, the underground part of the plant was harvested, and the rhizosphere soil and non-rhizosphere soil were mixed and filtered using a 40-mesh sieve to determine the physical and chemical properties of the soil.

### 2.4 Measurements

#### 2.4.1 Determination of *B. cereus* content

After the rhizosphere soil was screened by a 40 mesh sieve, the rhizosphere soil was collected and stored at −20°C for DNA extraction by kit (DNeasy ^®^PowerSoil Pro^®^ Kit, QIAGEN^®^, Germany). DNA concentration was measured using NanoPhotometer N60 spectrophotometer (IMPLEN, Germany), and the PCR primers for qRT-PCR analysis (Bio-Rad CFX96 touch, Bio-Rad Laboratories Incorporated, USA) were designed based on the sequence of *B. cereus* strains. The specific determination method referred to the existing research (Sun et al., 2022).

Initially, the plant roots were rinsed multiple times with distilled water, subsequently immersed in anhydrous ethanol, and finally pulverized using liquid nitrogen. DNA was extracted from plant roots using a kit (DNeasy ^®^ PowerPlant ^®^ Kit, QIAGEN ^®^, Germany). The DNA concentration was determined by NanoPhotometer N60 and quantified by qPCR.

#### 2.4.2 Analysis of soil bacterial community structure

Microbial community genomic DNA was extracted from the screened rhizosphere soil samples using the E.Z.N.A.^®^ soil DNA Kit (Omega Bio-tek, Norcross, GA, U.S.). The DNA extract was checked on 1% agarose gel, and DNA concentration and purity were determined with NanoDrop 2000 UV-vis spectrophotometer (Thermo Scientific, Wilmington, USA). The hypervariable region V3-V4 of the bacterial 16S rRNA gene was amplified with primer pairs 338F (5’-ACTCCTACGGGAGGCAGCAG-3’) and 806R(5’-GGACTACHVGGGTWTCTAAT-3’) by an ABI GeneAmp^®^ 9700 PCR thermocycler (ABI, CA, USA). The PCR amplification of 16S rRNA gene was performed as follows: initial denaturation at 95°C for 3 min, followed by 27 cycles of denaturing at 95°C for 30 s, annealing at 55°C for 30 s, and extension at 72°C for 45 s, and single extension at 72°C for 10 min, and end at 10°C. PCR reactions were performed in triplicate. The PCR product was extracted from 2% agarose gel and purified using the AxyPrep DNA Gel Extraction Kit (Axygen Biosciences, Union City, CA, USA) and quantified using Quantus™ Fluorometer (Promega, USA).

Purified amplicons were pooled in equimolar and paired-end sequenced on an Illumina MiSeq PE300 platform/NovaSeq PE250 platform (Illumina, San Diego, USA) according to the standard protocols by Majorbio Bio-Pharm Technology Co. Ltd. (Shanghai, China). The raw reads were deposited into the CNCB Genome Sequence Archive (GSA) database (GSA Accession Number: CRA016029).

The raw 16S rRNA gene sequencing reads were demultiplexed, quality-filtered by fastp version 0.20.0 (Chen et al., 2018) and merged by FLASH version 1.2.7 (Magoč and Salzberg, 2011) with the following criteria: (i) the 300 bp reads were truncated at any site receiving an average quality score of<20 over a 50 bp sliding window, and the truncated reads shorter than 50 bp were discarded, reads containing ambiguous characters were also discarded; (ii) only overlapping sequences longer than 10 bp were assembled according to their overlapped sequence. The maximum mismatch ratio of overlap region is 0.2. Reads that could not be assembled were discarded; (iii) Samples were distinguished according to the barcode and primers, and the sequence direction was adjusted, exact barcode matching, 2 nucleotide mismatch in primer matching.

Operational taxonomic units (OTUs) with 97% similarity cutoff were clustered using UPARSE version 7.1, and chimeric sequences were identified and removed (Stackebrandt and Goebel, 1994; Edgar, 2013). The taxonomy of each OTU representative sequence was analyzed by RDP Classifier version 2.2 against the 16S rRNA database using confidence threshold of 0.7 (Wang et al., 2007).

#### 2.4.3 Alpha diversity of soil microorganisms

Alpha diversity analysis was used to reflect the richness and diversity of bacterial communities in the rhizosphere soil of *A. adenophora* and *E. lindleyanum*. The following are the formulas for Shannon’s index (*H_shannon_*) and Simpson’s index (*D_simpson_*), which are employed to quantify species diversity:


$$H_{shannon}=-\sum_{i=1}^{S_{obs}}\frac{n_{i}}{N}\ln\frac{n_{i}}{N}$$

$$D_{simpson}=\frac{\sum_{i=1}^{S_{obs}}n_{i}\left(n_{i}-1\right)}{N\left(N-1\right)}$$

( $S_{obs}$ represents the actual observed OTU; $n_{i}$ represents the number of sequences contained in the ith OTU; *N* represents the number of all sequences).

#### 2.4.4 Soil characteristics

The soil pH was measured according to the glass electrode method by the pH meter (Niu et al., 2007). The drying method was then used to determine the soil moisture content (Samuelsson et al., 2006; Tian et al., 2021). Ammonium nitrogen (NH_4_^+^-N), nitrate nitrogen (NO_3_^–^N), and available phosphorus (AP) were measured by kits (Ltd. SATD-1-G, SXTD-1-G, ASXL-1-G, Suzhou Keming Biotechnology, China). Flame atomic absorption spectrometry is used to measure available Potassium (AK) (Yushmanov et al., 2007).

#### 2.4.5 *A.gossypii* population growth

Following the inoculation of *A.gossypii*, a cylindrical sleeve (perforated around) made of a PVC plastic plate covered each plant to prevent the *A.gossypii* from escaping. Five replicates were set up per treatment (3 *A.gossypii* densities × 3 soil treatments× 2 plant populations = 18 treatments).

The population of *A.gossypii* on each plant was recorded at three-day intervals throughout nine days, and the underground parts of the plant were harvested on the tenth day. When the insect’s body turns black and does not respond with a brush tip, it is considered that the insect is dead, and then delicately eliminated the deceased A.gossypii using a brush.

### 2.5 Statistical analysis

All data were analyzed by DPS 9.01 (Data Processing System v9.01) software. Before Analysis of variance (ANOVA) analysis, all data were evaluated to confirm that they conformed to the normal distribution; the data were the mean ± SE of five replicates per treatment. The effects of varying densities of *A.gossypii* (the number of *A.gossypii* was 0,5,10,15) and different plant species (*A. adenophora* and *E. lindleyanum*) on the *B. cereus* content in rhizosphere soil and soil characteristics were analyzed by two-way analysis of variance. The same approach was used to analyze the effects of varying additional treatments (CK\TC\BC) and different plant species on the population growth of *A.gossypii* and the characteristics of the soil. The difference between the two groups was compared by independent sample *t*-tests. The significant difference between treatments was based on the LSD test. P< 0.05 was considered statistically significant.

## 3 Results

### 3.1 Effects of *A.gossypii* feeding on *B. cereus* in rhizosphere soil and roots of *A. adenophora*

**Figure 1:**
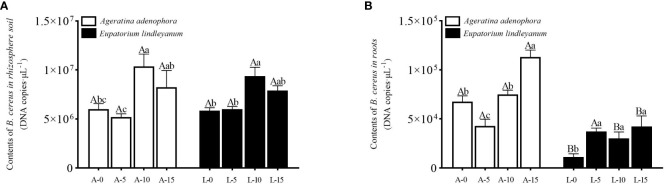
Variation of *B. cereus* content in root and rhizosphere soil for *A*. *adenophora* and *E. lindleyanum* treated by *A.gossypii*. **(A)** *B. cereus* content in the rhizosphere soil **(B)** *B. cereus* content in root; A represents *Ageratina adenophora*, L represents *Eupatorium lindleyanum*, 0/5/10/15 represents the number of *A.gossypii* inoculated, different lowercase letters represent significant difference (LSD’s test, P<0.05) at the same plant, different uppercase letters indicate significant differences between treatments with same *A. gossypii* infestation (t-test, P<0.05). Error Bars are mean ± SE (n=5).

The *B. cereus* content of the rhizosphere soil changed significantly at different *A.gossypii* densities within the same plants (*F*=10.8340, *P*<0.001, **Figure 1A**), but it did not differ between plants after *A.gossypii* feeding. In the treatment group of *A. adenophora*, the *B. cereus* content of the A-10 treatment increased by 72.58% compared with the A-0 treatment. Similarly, in the related species *E. lindleyanum*, the *B. cereus* content in the L-10 treatment group was 58.65% higher than that in the L-0 treatment group (**Figure 1A**). This indicated that the *A.gossypii* infestation promoted the increase of *B. cereus* content in the rhizosphere soil of *A. adenophora* and *E. lindleyanum*.

On the other hand, changes in the *B. cereus* content in *A. adenophora* roots differ from those in rhizosphere soil. There are significant differences in different plants (*F*=86.080, *P*<0.001, **Figure 1B**) and different *A.gossypii* densities (*F*= 13.619, *P*<0.001, **Figure 1B**), as well as interactions between them (*F*= 8.869, *P*<0.001, **Figure 1B**). The A-15 treatment group had significantly higher *B. cereus* content than the other treatments, and the *B. cereus* content in the *A. gossypii* treatment group in *E. lindleyanum* was higher than in the treatment group without an *A. gossypii* infestation(**Figure 1B**). Within the same *A.gossypii* density group, except for the treatment with inoculation *A.gossypii* density of 5, the content of *B. cereus* in the roots of *A. adenophora* increased by 500.64% compared with that of *E. lindleyanum* in other treatment groups (**Figure 1B**), indicating that *A.gossypii* feeding could significantly increase the *B. cereus* content in the roots of *A. adenophora* compared to its local relatives.

### 3.2 Effects of *A.gossypii* feeding on physical and chemical properties of *A.adenophora*’s rhizosphere soil

**Figure 2:**
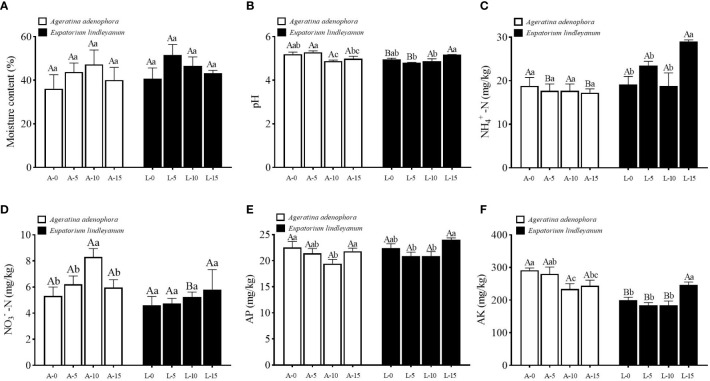
Physicochemical characteristic changes in rhizosphere soil for two kinds of plants treated with *A.gossypii*. **(A)** moisture content. **(B)** pH value. **(C)** NH_4_^+^-N, ammonium nitrogen. **(D)** NO_3_^–^N, nitrate nitrogen. **(E)** AP, available phosphorus. **(F)** AK, available potassium. A represents *Ageratina adenophora*, L represents *Eupatorium lindleyanum*, 0/5/10/15 represents the number of *A.gossypii* inoculated, different lowercase letters represent significant difference(LSD’s test, P<0.05) at the same plant, different uppercase letters indicate significant differences between treatments with same *A*. *gossypii* infestation (*t*-test, P<0.05). Error Bars are mean ± SE (n=5).

The soil’s physical and chemical properties were measured after *A.gossypii* feeding on two plants. In terms of soil moisture content, the results showed that *A.gossypii* feeding at varied densities and plants had no significant effect on it (**Figure 2A**). Nonetheless, the pH of the soil can be greatly impacted by varying A. gossypii feeding densities. Compared with the A-0 treatment, pH decreased by 6.15% in the A-10 treatment, and there was a significant interaction between different plants and different *A.gossypii* densities (*F*=7.537, *P*<0.001, **Figure 2B**). NH_4_^+^-N in rhizosphere soil of *A. adenophora* did not vary significantly across *A.gossypii* densities, but it varied widely and was significantly higher in *E. lindleyanum* compared to *A. adenophora* at *A.gossypii* densities of 5 or 15 (**Figure 2C**). NO_3_^–^N content of *A. adenophora* differed significantly under different treatments (*F*=5.273, *P*<0.05, **Figure 2D**). Specifically, the content of NO_3_^–^N in the A-10 treatment increased by 33.72% compared to the A-0 treatment, whereas there was no significant change observed in the control *E. lindleyanum* (**Figure 2D**). The content of AP in the rhizosphere soil of the two plants did not significantly differ when exposed to different densities of *A.gossypii* treatments (**Figure 2E**). However, in *A. adenophora*, it decreased significantly by 13.86% in the A-10 treatment as compared to the A-0 treatment (**Figure 2E**). *A.gossypii* feeding at different densities (*F*=38.002, *P*<0.001, **Figure 2F**) and plants (*F*=3.158, *P*<0.05, **Figure 2F**) significantly affected soil AK content, and there occurred significant interactions between different plants and *A.gossypii* densities(*F*=5.786, *P*<0.001, **Figure 2F**). The content of AK decreased to 19.56% and 16.29% in the A-10 and A-15 treatments, respectively, compared to the A-0 treatment (**Figure 2F**).

### 3.3 Effects of *A.gossypii* feeding on soil bacterial community structure

**Figure 3:**
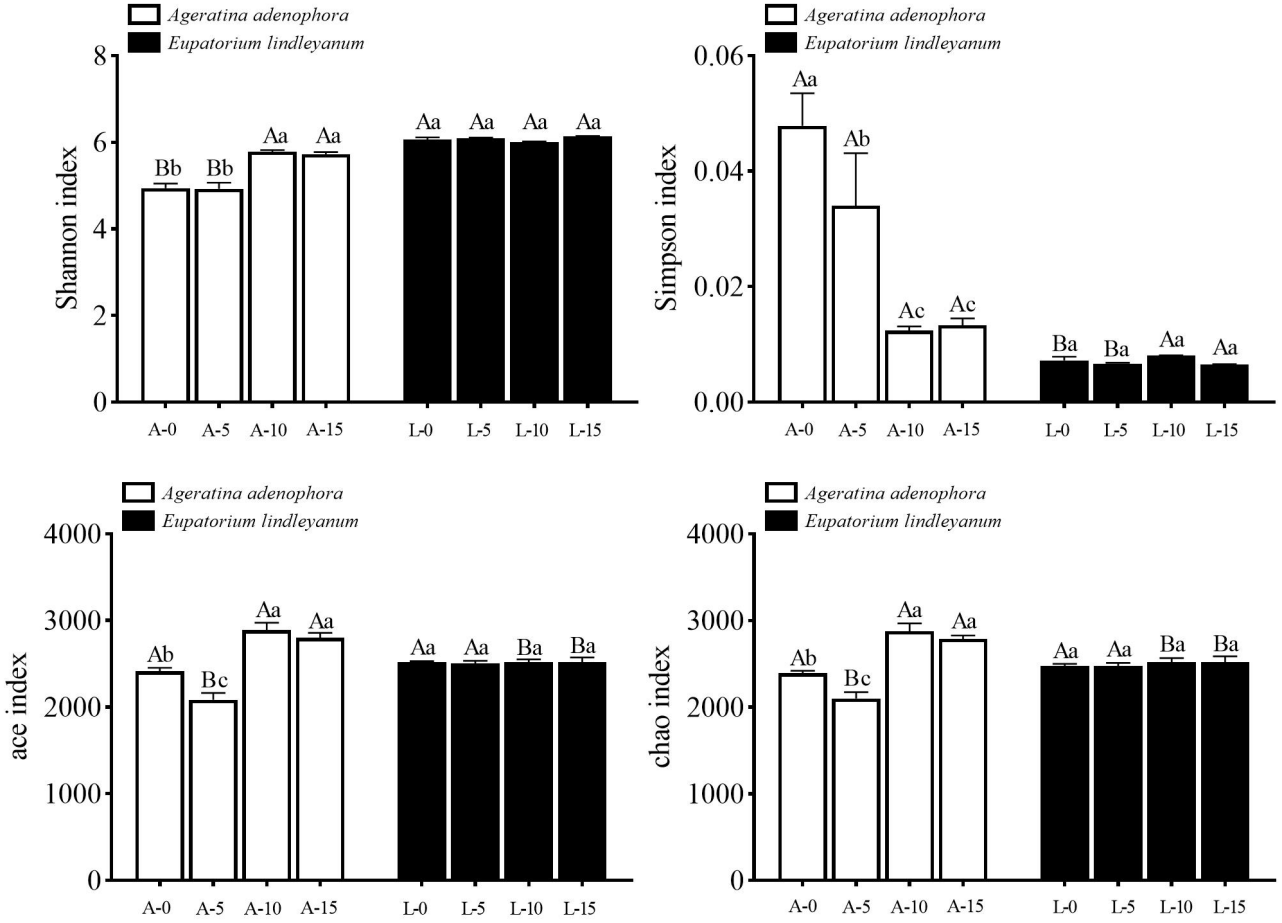
Changes of Alpha diversity indices for bacterial communities in rhizosphere soil for two kinds of plants treated with *A.gossypii*. A represents *Ageratina adenophora*, L represents *Eupatorium lindleyanum*, 0/5/10/15 represents the number of *A.gossypii* inoculated, different lowercase letters represent significant difference(LSD’s test, P<0.05) at the same plant, different uppercase letters indicate significant differences between treatments with same *A. gossypii* infestation (*t-test*, P<0.05). Error Bars are mean ± SE (n=5).

Different plants and densities of *A.gossypii* feeding considerably altered both the Shannon and Simpson indexes, with a significant interaction between the two parameters (Shannon index: *F*= 216.661, *P*<0.001; *F*=22.845, *P*<0.001; *F*= 23.983, *P*<0.001; Simpson index: *F*= 53.609, *P*<0.001; *F*= 9.979, *P*<0.001; *F*=10.261, *P*<0.001). However, the ace, chao index could be significantly changed only under different *A.gossypii* density treatments (ace index: *F*=24.555, *P*<0.001; *F*=22.831, *P*<0.001; chao index: *F*=23.160, *P*<0.001; *F*=17.770, *P*<0.001). In *A. adenophora*, the Shannon index of the rhizosphere soil bacterial community was significantly higher in treatment A-10 along with treatment A-15 than in treatment A-0 together with treatment A-5, and there was no difference in Shannon index was observed among treatment groups in the control plant *E. lindleyanum*, and there was no difference in Shannon index among treatment groups in the control plant, *E. lindleyanum* (**Figure 3**). The composition of the rhizosphere soil bacterial community showed the same trend in Simpson’s index for both plants, with a significant decrease of 74.11% in Simpson’s index for A-10 compared to A-0 (**Figure 3**). Meanwhile, the ace index and chao index of the A-5 treatment were remarkably lower from other treatments, and a significant difference was observed between the A-5 treatment and the L-5 treatment, and the A-10 treatment and the A-15 treatment had a significantly higher ace index and chao index than the L-10 treatment and the L-15 treatment (**Figure 3**).

**Figure 4:**
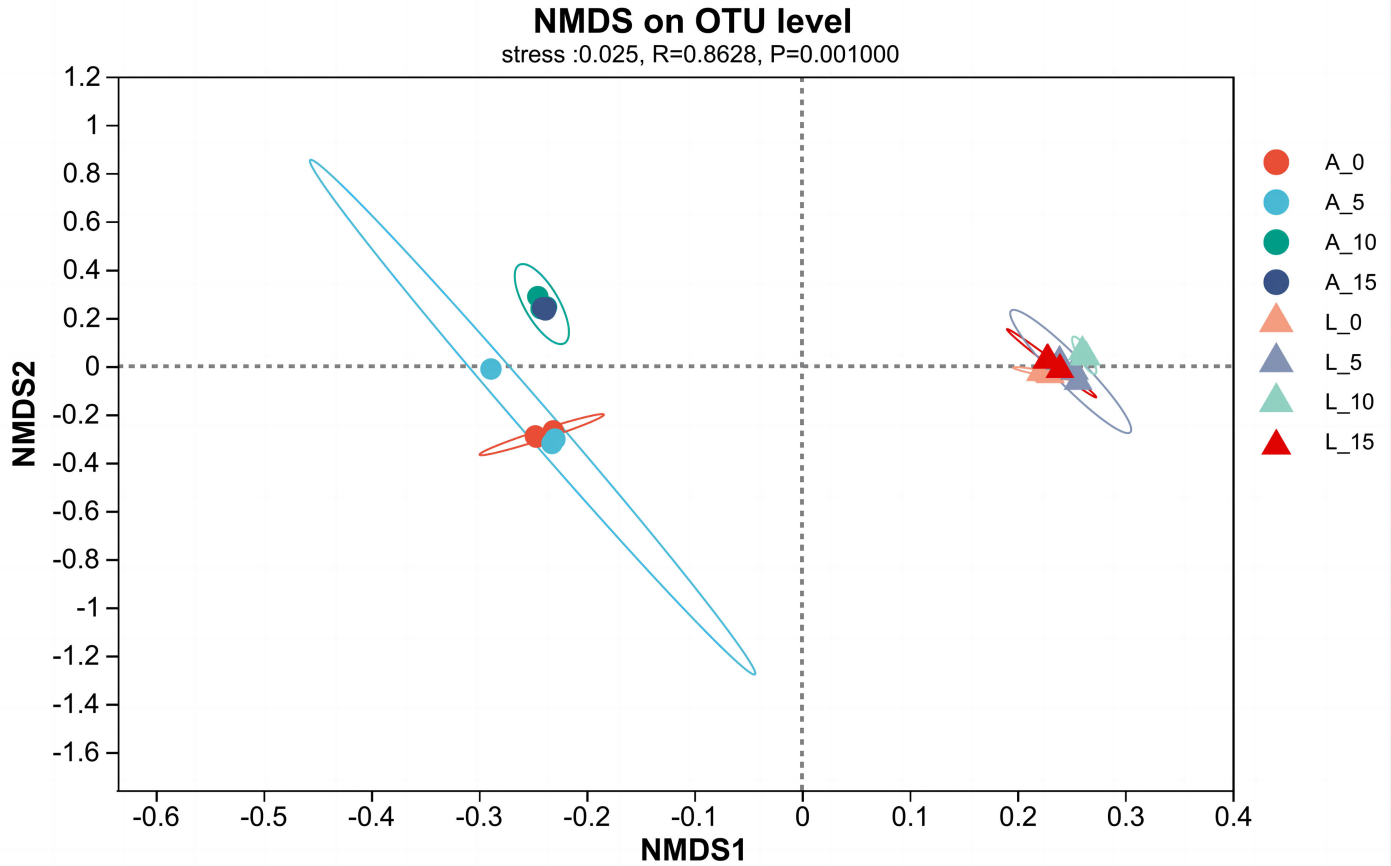
Non-metric multidimensional scaling of β diversity in soil bacterial communities after *A.gossypii* feeding. A represents *Ageratina adenophora*, L represents *Eupatorium lindleyanum*, 0 represents no A.gossypii, 5 represents 5 nymphs, 10 represents 10 nymphs, and 15 represents 15 nymphs.

Based on the original OTUs of the plant rhizosphere soils, we carried out non-metric multidimensional scaling (NMDS), which reflects the species composition in each sample on a multidimensional space in the form of points, and the greater the point-to-point distance, the greater the sample differences. As shown in **Figure 4**, there were significant differences between the soil bacterial communities of the two plants, *A. adenophora*, and *E. lindleyanum*, under the same density of *A.gossypii* feeding (**Figure 4**). Interestingly, *A.gossypii* feeding increased the β-diversity of the soil bacterial community in *A. adenophora*, while there was no increase in β-diversity in the soil bacterial community of *E. lindleyanum* after *A.gossypii* inoculation, suggesting that the two plants have different changes in rhizosphere soil microbial communities for the various densities of *A.gossypii* feeding (**Figure 4**).

### 3.4 Effects of *A.gossypii* feeding on the number of bacterial OTUs in the rhizosphere soil of *A.adenophora*

**Figure 5:**
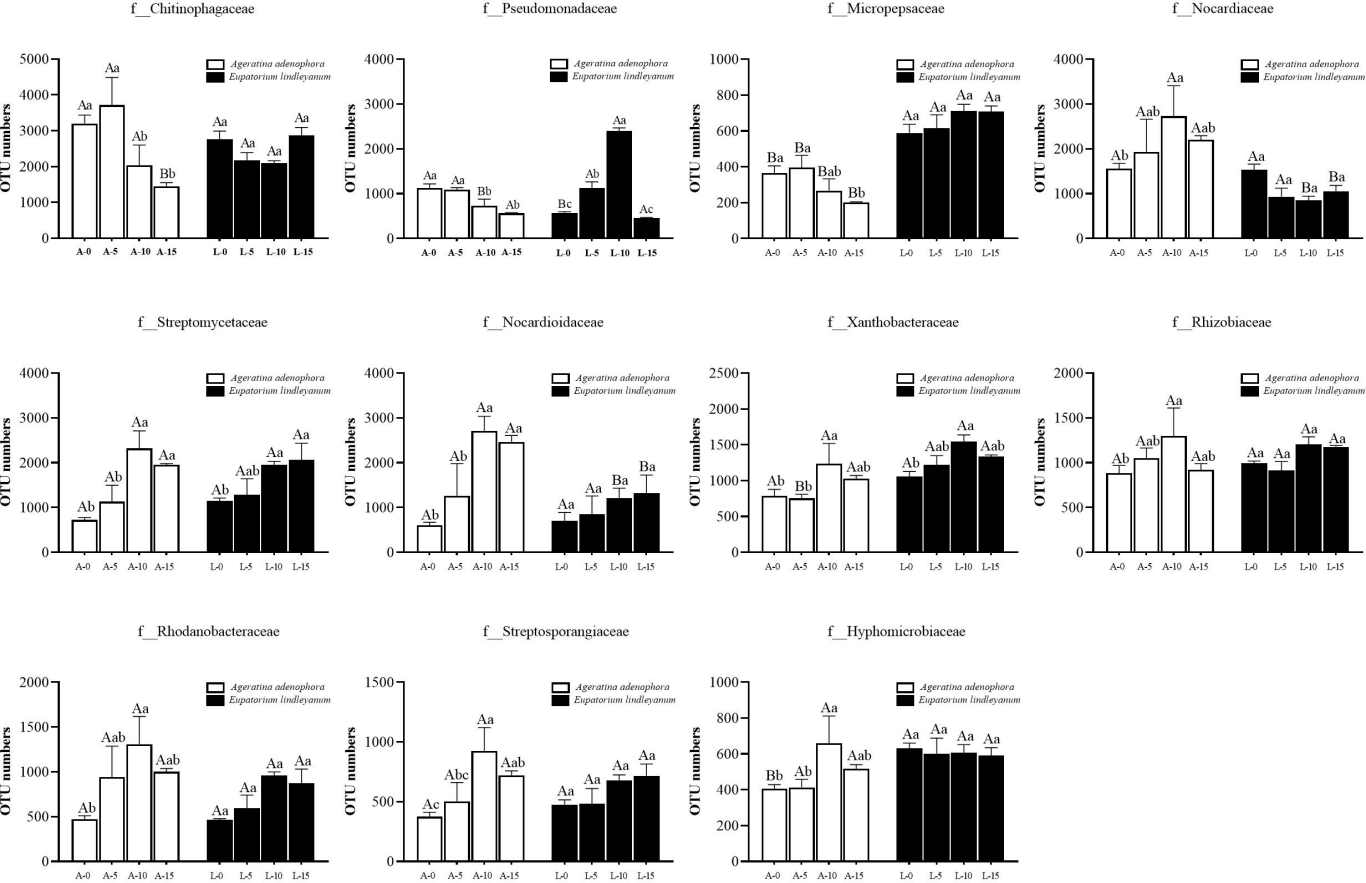
Changes of bacteria in *A. adenophora* with rhizosphere soil OTU numbers under *A. gossypii* feeding at the family level. A represents *Ageratina adenophora*, L represents *Eupatorium lindleyanum*, 0/5/10/15 represents the number of *A.gossypii* inoculated, different lowercase letters represent significant difference (LSD’s test, P<0.05) at the same plant, different uppercase letters indicate significant differences between treatments with same *A. gossypii* infestation (LSD’s test, P<0.05). Error Bars are mean ± SE (n=5).

To further explore the relationship between changes in bacterial communities and *A.gossypii* feeding, we obtained 11 families of bacteria in the rhizosphere soil of *A. adenophora* that responded to *A.gossypii* feeding by sequencing. The number of OTUs represents the abundance of bacteria. As shown in **Figure 5**, the bacterial abundance of three families in the rhizosphere soil of *A. adenophora* decreased significantly, namely *Chitinophagaceae*, *Pseudomonadaceae*, *Micropepsaceae*, and compared with the L-10 treatment, the OTU number of *Pseudomonadaceae* was significantly increased in the A-10 treatment; the OTU number of *Micropepsaceae* was considerably higher than the control treatment in all treatments (**Figure 5**).

The abundance of 8 bacterial families has increased: *Nocardiaceae*, *Streptomycetaceae*, *Nocardioidaceae*, *Xanthobacteraceae*, *Rhizobiaceae*, *Rhodanobacteraceae*, *Streptosporangiaceae*, and *Hyphomicrobiaceae* (**Figure 5**). At a feeding density of 10 nymphs, the number of OTU number of *Streptomycetaceae*, *Nocardioidaceae*, *Xanthobacteraceae*, and *Streptosporangiaceae* were all significantly higher than those of treatment groups with only 5 or no *A.gossypii* feeding (**Figure 5**). In addition, the OTU number of *Nocardiaceae* and *Nocardioidaceae* in the rhizosphere soil of *A. adenophora* at the same feeding density (10) was significantly increased by 2.19 and 1.23-folds compared to that of the local relative, *E. lindleyanum* (**Figure 5**). Interestingly, the conclusion we emerge from the comparison of the data is that *A.gossypii* feeding at different densities had no significant effect on the abundance of bacteria in the rhizosphere of *E. lindleyanum*, except for the three bacteria families, *Pseudomonadaceae*, *Streptomycetaceae*, and *Xanthobacteraceae* (**Figure 5**).

### 3.5 Effects of *B. cereus* on the population growth of *A. gossypii* at different initial densities

**Figure 6:**
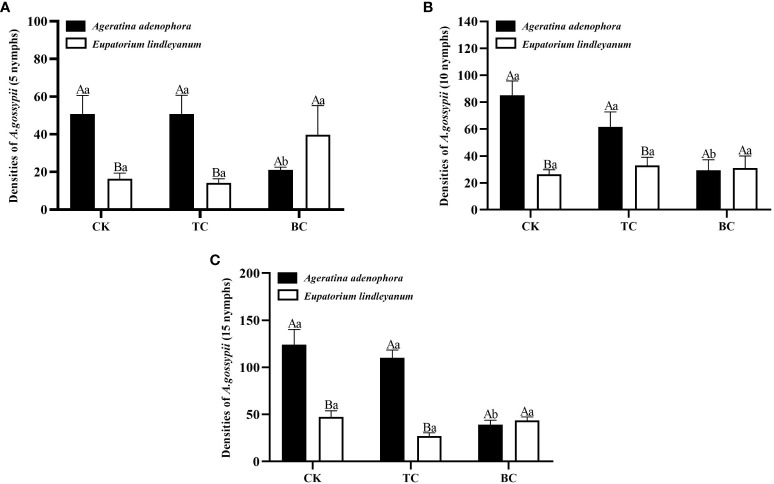
The population growth of *A. gossypii* on *A*. *adenophora* and *E*. *lindleyanum* after adding or excluding *B*. *cereus*. CK, addition with sterile water; TC, addition with thiosen copper; BC, inoculation with *B*. *cereus.* **(A)** represents infestation with 5 nymphs; **(B)** represents infestation with 10 nymphs; **(C)** represents infestation with 15 nymphs. Different lowercase letters above the bars represent significant differences among the three addition treatments (LSD’s test, P<0.05) in the same plant; different uppercase letters indicate significant differences between the same treatments in the different plants (*t*-test, P<0.05). Error Bars are mean ± SE (n=5).

The number of *A. gossypii* population growth on each treatment was counted on the 10th day after inoculation, and the results of ANOVA showed that the addition of *B. cereus* significantly changed the number of *A. gossypii* populations at initial inoculation numbers of 10 and 15 (*F*=4.784, *P*<0.05; *F*=13.070, *P*<0.001; **Figures 6B, C**) and that there was a significant interaction between inoculation with *B. cereus* and different plant sources (*F*=6.416, *P*<0.001; *F*=13.749, *P*<0.001, **Figures 6B, C**); and the growth of *A. gossypii* population was mainly associated with plant source when the initial number of *A. gossypii* was 5 (*F*=6.599, *P*<0.05; **Figure 6A**).

For *A. adenophora*, the addition of thiosen copper treatment showed no significant difference in the number of *A. gossypii* populations with initial inoculation of 5,10, and 15 on the 10th day compared to the sterile water treatment, as well as the addition of *B. cereus*, which resulted in significant reductions of *A. gossypii* populations by 58.66% (**Figure 6A**), 65.49% (**Figure 6B**), and 68.39% (**Figure 6C**), respectively, when compared to the sterile water control. For *E. lindleyanum*, the growth of the *A. gossypii* population on plants with thiosen copper and with *B. cereus* was not different from the control under three different *A. gossypii* feeding density treatments. Meanwhile, the *A. gossypii* population of *A. adenophora* was significantly higher than that of *E. lindleyanum* when no B. cereus was added compared to both plants (**Figure 6**).

### 3.6 Effects of addition with *B. cereus* on soil characteristics of *A. adenophora* fed by *A. gossypii*

**Figure 7:**
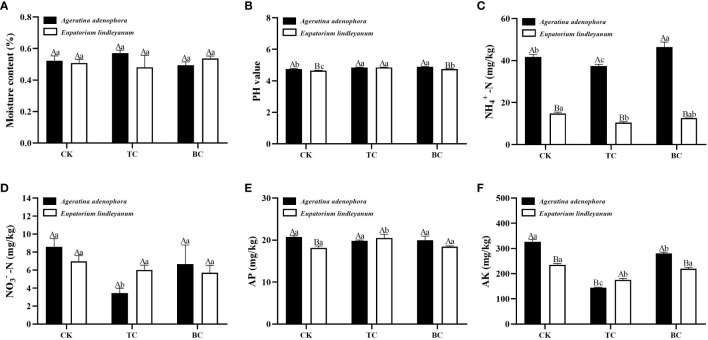
The changes in physicochemical characteristics of rhizosphere soil for two kinds of plants after being fed on by *A*. *gossypii* (15 nymphs) with the addition of *B.cereus*. **(A)** moisture content. **(B)** pH value. **(C)** NH_4_^+^-N, ammonium nitrogen. **(D)** NO_3_^–^N, nitrate nitrogen. **(E)** AP, available phosphorus. **(F)** AK, available potassium. CK, addition with sterile water; TC, addition with thiosen copper; BC, inoculation with *B*. *cereus.* Lowercase letters above the bars represent significant differences among the three addition treatments (LSD’s test, P<0.05) in the same plant, and different uppercase letters indicate significant differences between the same treatments in the different plants (t-test, P<0.05). Error Bars are mean ± SE (n=5).

Since the addition of *B. cereus* at an initial density of 15 *A. gossypii* inhibited the population growth of *A. gossypiis* more strongly, the soil physicochemical properties were measured by taking the rhizosphere soil of each plant of this treatment. Except for moisture content and AP, all soil properties varied substantially in soils treated with thiosen copper or *B.cereus* (**Figure 7**). The addition of *B. cereus* significantly increased the pH value of the rhizosphere soil of *A. adenophora* compared to the control (**Figure 7B**), and also increased the NH_4_^+^-N content by 10% (**Figure 7C**), while the content of AK decreased significantly by 14.29% (**Figure 7F**), and both of them was higher than that of the native plant, *E. lindleyanum*. Further data analysis revealed that after adding thiosen copper, the pH value increased compared to the CK. Additionally, the contents of NH_4_^+^-N, NO_3_^–^N, and AK significantly decreased by 10.30%, 59.70%, and 55.95%, respectively (**Figures 7C, D, F**). It was observed that the NH_4_^+^-N content in the rhizosphere soil of *E. lindleyanum* was significantly lower than that of *A. adenophora*, while the NO_3–_N and AK content were higher.

## 4 Discussion

Many plants possess the capacity to induce defenses (Rabari et al., 2023). When they are consumed by animals or insects, they increase the synthesis of secondary metabolites or accumulate microorganisms that are beneficial to their growth and development, thus strengthening their defenses (Miller et al., 2005). Our research investigated the beneficial bacteria in the rhizosphere environment of *A.adenophora* that responded to the action of broad-feeding natural enemies and the changes in the adaptability of A.adenophora under the interaction with them. The results showed that *A. gossypii* feeding on *A.adenophora* changed the content of *B.cereus* in both the rhizosphere soil and the root, and it was significantly increased when the number of *A.gossypii* reached 15 (**Figure 1**), which was consistent with the results of previous study on that in the generalist natural enemy, *procecidochares utilis* (Sun and Yang, 2021). It was found that the parasitism of *procecidochares utilis* significantly reduced the biomass of *A.adenophora*, and it counteracted this damage by accumulating the beneficial bacterium *B.cereus* (Erasmus et al., 1992). It demonstrates that to cope with the feeding of natural enemies, *A.adenophora* can improve its competitiveness in the environment by gathering *B.cereus*, which is beneficial to itself. Compared with *A.adenophora*, the native relative species *E.lindleyanum* did not have such characteristics.

In terms of soil physicochemical properties, we discovered that pH, AP, and AK contents of the rhizosphere soil of *A.adenophora* decreased after *A. gossypii* feeding (**Figures 2B, E, F**), whereas NO_3_^–^N increased significantly (**Figure 2D**). The metabolism of microorganisms and animals in the rhizosphere of plants directly promotes or inhibits the absorption and growth of nutrients in the roots (Adedayo and Babalola, 2023) and can also affect the transformation of N, P, K, and other substances in the rhizosphere soil (Piton et al., 2023). Relevant studies have shown that the soil characteristics changed after the invasion of *A.adenophora*, especially the contents of NO_3_^–^N, NH_4_^+^-N, AK, and AP in the soil increased (Zhao et al., 2019). In this study, it was found that the AK content in the rhizosphere soil of *A.adenophora* under *A.gossypii* feeding was higher than that of *E.lindleyanum*. Furthermore, *A.adenophora* was found to accumulate *B.cereus*, which is a kind of growth-promoting bacteria to benefit itself (Chen et al., 2019; Sun et al., 2021). The experimental results revealed the *B. cereus* mediated defense of *A.adenophora* against natural enemies.

α diversity refers to the species diversity among individuals within a specific geographic area or ecosystem (Thukral, 2017). Among them, the Chao1 index and ACE index can be used to describe the species abundance of bacteria, while the Shannon index and Simpson index were used to measure the diversity of bacterial community (Xia and Sun, 2023). Compared with the Simpson index, the Shannon index comprehensively takes into account the evenness and richness of the community, and the higher the value is, the greater the diversity is (Kumar et al., 2023). Affected by the species abundance and species evenness in the sample community, under the same species abundance condition, if the species in the community are relatively uniform, the diversity of the community is higher (Kimbro and Grosholz, 2006). Our investigation revealed that there were no significant differences in α-diversity across the treatment groups following the feeding of *A.gossypii* at various densities on *E.lindleyanum*.

Nevertheless, The α-diversity of *A.adenophora* changed significantly. Among the four types of indexes, there were significant differences between the treatment with *A.gossypii* density of 10, the treatment with *A.gossypii* density of 15, and the blank control, respectively. There was also a significant difference between the two plants when they were fed by the same *A.gossypii* density (10 and 15; **Figure 3**). According to the Shannon index, the diversity of each treatment of *A.adenophora* was significantly lower than that of *E.lindleyanum* (**Figure 3**), which was consistent with the research results that invasive organisms could greatly decrease the α-diversity of soil microbial communities (Stefanowicz et al., 2021). At the same time, the Shannon index of the rhizosphere soil bacterial community of *A.adenophora* increased significantly under the feeding of *A.gossypii*, while that of *E.lindleyanum* did not change significantly (**Figure 3**), indicating that *A.adenophora* had a stronger ability to change its bacterial community under the feeding of natural enemies.

β diversity is commonly defined as a consistent change in species between different locations, which provides local-scale biodiversity and a broader regional species pool (Anderson et al., 2011). Non-Metric Multi-Dimensional Scaling (NMDS) is a ranking technique used in β-diversity analysis, specifically in ecological research (Zhao et al., 2020). The results of NMDS analysis revealed that the bacterial structure in the rhizosphere soil of *A.adenophora* and *E.lindleyanum* was significantly different when they were not inoculated with *A.gossypii* (**Figure 4**). Moreover, the rhizosphere soil bacterial community of *A.adenophora* with 10 and 15 aphids feeding was significantly changed compared with the blank control and 5 aphids feeding treatments (**Figure 4**), while that of *E.lindleyanum* was not, which as previously described that *A.adenophora* can selectively accumulate bacteria that are advantageous to its own growth (Chen et al., 2019). The bacteria accumulated in the rhizosphere soil showed a specific growth-promoting effect on the plants (Cunha et al., 2024). What’s more. Increases in soil bacterial populations and changes in species structure can provide a competitive advantage for *A.adenophora* after invasion (Fang et al., 2019). Our results illustrate the conclusion that *A.adenophora* has a stronger ability to change its bacterial community in response to natural enemy feeding.

Then we continued to analyze the effect of *A.gossypii* feeding on the number of bacterial OUT in *A.adenophora’s* rhizosphere soil by sequencing and summarizing the bacterial species with significant changes in abundance at the family level (**Figure 5**). Among the 11 bacterial families with significant changes, *Xanthobacteraceae*, *Rhizobiaceae*, and *Hyphomicrobiaceae*, which belong to the order *Rhizobiales*, showed a significant increase in their OTU numbers in the rhizosphere soil of *A.adenophora* after inoculation with 10 aphids. It was reported that the family *Xanthobacteraceae* has included many aerobic chemoheterotrophic genera, which contribute to N_2_ fixation (Ma et al., 2019). *Rhizobaceae* can provide nitrogen fertilizer for plants and increase the nitrogen content in soil (Sprent et al., 2017), and *Hyphomicrobiaceae is a family of photosynthetic bacteria in Rhizobium* (Lian et al., 2021). Nitrogen is an indispensable substance for plant growth and is the main source of protein synthesis (Li et al., 2023). Nitrogen-fixing bacteria can provide it for plants through nitrogen fixation, thereby promoting plant growth and development (Masson-Boivin and Sachs, 2018), and the increase in the abundance of these bacteria is positively correlated with the results of our previous study that found an increase in NO_3_^–^N content.

Due to the phenotypic plasticity, compensatory or tolerance of invasive species, the negative feedback effect is significantly weakened (Policelli et al., 2018). On the other hand, some rhizosphere-beneficial bacteria have a preferential impact on invasive species (Sun et al., 2022). For instance, mycorrhizal fungi significantly improve the disease resistance of host plants, and the invasive plant *Anthemis cotula* is protected from the invasion of pests and diseases due to the high infection of arbuscular mycorrhizal fungi (Shah et al., 2008). Our research has revealed that *A.gossypii* feeding can stimulate the increase of *B. cereus* content in rhizosphere soil. Therefore, we hypothesize that the addition of B.cereus will affect the growth of the *A.gossypii* population as well as the soil characteristics. The results showed that the growth rate of the *A.gossypii* population was significantly slowed down after the addition of B. cereus to *A.adenophora* (**Figure 3C**). In the native plant *E.lindleyanum*, the addition of *B. cereus* did not impede the growth of *A.gossypii* population, so it can be speculated that the addition of *B.cereus* enhanced the resistance of *A.adenophora*, which is consistent with the previous results of Du et al. (2022b) found that the survival and reproduction of *A.gossypii* inoculated with arbuscular mycorrhizal fungi on *A.adenophora* were inhibited. This could be a result of the *A.gossypii* being poisoned by the leaves’ elevated concentration of insect-resistant chemicals (Zhu et al., 2024).

In the determination of soil physical and chemical properties, we found that even in the case of *A.gossypii* feeding, the addition of *B.cereus* significantly increased the content of NH_4_^+^-N in the rhizosphere soil of *A.adenophora*, but this effect was not obvious in *E.lindleyanum* (**Figure 7C**). NH_4_^+^-N is one of the forms of nitrogen that can be absorbed and utilized by plants (Liu and Von Wirén, 2017). Studies have shown that *B. cereus* can promote the accumulation of nitrogen in plants (Vessey and Buss, 2002), which is consistent with our experimental results. In addition, the results of this study indicated that the two plants have different nitrogen utilization, and also suggested that the growth-promoting effect or resistance improvement of *B.cereus* on *A.adenophora* may be achieved by increasing the ammonium nitrogen in the soil.

In summary, compared to local closely related species, *A. gossypii* feeding significantly increased the content of *B. cereus* in the rhizosphere and in the roots of *A. adenophora*, induced remarkable changes in the α-diversity and β-diversity of rhizosphere bacterial communities, along with an increase in NO_3_^–^N content in the rhizosphere soil, which confirms that *A. adenophora* can exert a biased impact on the rhizosphere soil environment under the influence of a generalist predator. The *B. cereus* addition/exclusion test revealed that an elevated concentration of *B. cereus* content in the soil could inhibit the population growth of *A. gossypii* on *A. adenophora*. Apart from that, the NH_4_^+^-N content in the rhizosphere soil also enhanced, suggesting that the increase of *B. cereus* content provided positive assistance in helping *A. adenophora* resist insect feeding. We investigated the role of *B. cereus* in the adaptation of *A. gossypii* to a wide range of predatory natural enemies from the perspective of the bidirectional role of natural enemies and inter-root beneficial bacteria, and the further research will explore how *B. cereus* activates the JA and SA pathways in *A. adenophora* from the viewpoint of triggering induced systemic resistance (ISR) by beneficial rhizosphere bacteria, and analyze the molecular mechanisms by which beneficial bacteria enhance the adaptability of *A. adenophora* to predators, laying the foundation for evaluating the biological control effect of *A. gossypii* on *A. adenophora*.

## Data availability statement

The original contributions presented in the study are included in the article/**Supplementary Material**, and https://bigd.big.ac.cn/gsa/browse/CRA016029, accession number: CRA016029. further inquiries can be directed to the corresponding author/s.

## Ethics statement

The manuscript presents research on animals that do not require ethical approval for their study.

## Author contributions

YY: Data curation, Writing – original draft, Writing – review & editing, Formal Analysis, Investigation. ZY: Data curation, Investigation, Writing – original draft, Formal Analysis, Methodology. MH: Data curation, Investigation, Writing – original draft. SS: Conceptualization, Methodology, Writing – review & editing. GX: Conceptualization, Writing – review & editing, Methodology. GY: Conceptualization, Funding acquisition, Writing – review & editing, Methodology.
