# Supplementary material for: Beneficial rhizosphere bacteria provides active assistance in resisting Aphis gossypiis in Ageratina adenophora
Source: Front Plant Sci. 2024 May 15;15:1394153. doi: 10.3389/fpls.2024.1394153 (PMC11133562; doi:10.3389/fpls.2024.1394153)
Supplement: Supplementary file 1 [file Table_1.docx]

Supplementary Material

Beneficial rhizosphere bacteria provides active assistance in resisting *A.gossypii*s in *Ageratina adenophora*

Youxin Yu^1^, Zihao Yang^1^, Mengyang Han^1^, Shengnan Sun^2^, Gang Xu^1^, Guoqing Yang^1^*

^1^College of Plant Protection, Yangzhou University,Yangzhou, China

^2^College of Animal Science and Technology, Yangzhou University, Yangzhou, China

*** Correspondence:** Guoqing Yang [gqyang@yzu.edu.cn](mailto:gqyang@yzu.edu.cn)

# Supplementary Figures and Tables

## Supplementary Tables

**Supplementary Tables 1.** Two-way ANOVA of the effects of *A.gossypii* feeding on *B. cereus* content in rhizosphere soil and roots of *A. adenophora.*

|  | Variables | F | P |
| --- | --- | --- | --- |
| roots | species | 86.080 | < 0.0001*** |
|  | *A. gossypii* | 13.619 | < 0.0001*** |
|  | species**A. gossypii* | 8.869 | < 0.0001*** |
| rhizosphere soil | species | 0.032 | 0.8583 |
|  | *A. gossypii* | 9.783 | <0.0001*** |
|  | species**A. gossypii* | 0.281 | 0.8388 |

**Supplementary Tables 2.** Two-way ANOVA of the effects of *A.gossypii* feeding on physical and chemical properties of rhizosphere soil of *A.adenophora*

|  | Variables | F | P |
| --- | --- | --- | --- |
| Moisture content | species | 1.083 | 0.3085 |
|  | *A. gossypii* | 1.516 | 0.2358 |
|  | species**A. gossypii* | 0.241 | 0.8670 |
| pH | species | 6.821 | 0.0136* |
|  | *A. gossypii* | 3.562 | 0.0249* |
|  | species**A. gossypii* | 7.537 | 0.0006*** |
| NH_4_^+^-N | species | 12.868 | 0.0006*** |
|  | *A. gossypii* | 2.248 | 0.0903 |
|  | species**A. gossypii* | 4.665 | 0.0050 ** |
| NO_3_^–^-N | species | 5.273 | 0.0231* |
|  | *A. gossypii* | 1.486 | 0.2210 |
|  | species**A. gossypii* | 1.507 | 0.2152 |
| AP | species | 1.713 | 0.1949 |
|  | *A. gossypii* | 4.809 | 0.0042** |
|  | species**A. gossypii* | 1.235 | 0.3035 |
| AK | species | 38.002 | 0.0001*** |
|  | *A. gossypii* | 3.158 | 0.0380* |
|  | species**A. gossypii* | 5.786 | 0.0028** |

**Supplementary Tables 3.** Two-way ANOVA of the effects of *A.gossypii* feeding on soil bacterial community structure

|  | Variables | F | P |
| --- | --- | --- | --- |
| Shannon index | species | 216.661 | <0.0001*** |
|  | *A. gossypii* | 22.845 | <0.0001*** |
|  | species**A. gossypii* | 23.983 | <0.0001*** |
| Simpson index | species | 53.609 | <0.0001*** |
|  | *A. gossypii* | 9.979 | 0.0006*** |
|  | species**A. gossypii* | 10.261 | 0.0005*** |
| ace index | species | 0.732 | 0.4048 |
|  | *A. gossypii* | 24.555 | <0.0001*** |
|  | species**A. gossypii* | 22.831 | <0.0001*** |
| chao index | species | 1.062 | 0.3181 |
|  | *A. gossypii* | 23.160 | <0.0001*** |
|  | species**A. gossypii* | (Xia, 2023 #131) | <0.0001*** |

**Supplementary Tables 4.** Two-way ANOVA of the effects of *A.gossypii* feeding on soil bacterial community structure

|  | Variables | F | P |
| --- | --- | --- | --- |
| Chitinophagaceae | species | 0.217 | 0.6477 |
|  | *A. gossypii* | 3.408 | 0.0433* |
|  | species**A. gossypii* | 5.267 | 0.0102* |
| Pseudomonadaceae | species | 18.101 | 0.0006*** |
|  | *A. gossypii* | 52.997 | <0.0001*** |
|  | species**A. gossypii* | 64.086 | <0.0001*** |
| Micropepsaceae | species | 92.478 | <0.0001*** |
|  | *A. gossypii* | 0.344 | 0.7940 |
|  | species**A. gossypii* | 4.250 | 0.0218* |
| Nocardiaceae | species | 14.999 | 0.0013** |
|  | *A. gossypii* | 0.333 | 0.8013 |
|  | species**A. gossypii* | 2.127 | 0.1369 |
| Streptomycetaceae | species | 0.176 | 0.6805 |
|  | *A. gossypii* | 9.721 | 0.0007*** |
|  | species**A. gossypii* | 0.738 | 0.5445 |
| Nocardioidaceae | species | 8.252 | 0.0110* |
|  | *A. gossypii* | 6.227 | 0.0053** |
|  | species**A. gossypii* | 1.951 | 0.1622 |
| Xanthobacteraceae | species | 14.747 | 0.0014** |
|  | *A. gossypii* | 5.653 | 0.0078** |
|  | species**A. gossypii* | 0.253 | 0.8584 |
| Rhizobiaceae | species | 0.122 | 0.7312 |
|  | *A. gossypii* | 2.187 | 0.1294 |
|  | species**A. gossypii* | 0.974 | 0.4292 |
| Rhodanobacteraceae | species | 2.564 | 0.1289 |
|  | *A. gossypii* | 4.779 | 0.0146* |
|  | species**A. gossypii* | 0.426 | 0.7370 |
| Streptosporangiaceae | species | 0.292 | 0.5964 |
|  | *A. gossypii* | 5.415 | 0.0092** |
|  | species**A. gossypii* | 0.891 | 0.4672 |
| Hyphomicrobiaceae | species | 4.897 | 0.0418* |
|  | *A. gossypii* | 1.363 | 0.2896 |
|  | species**A. gossypii* | 1.614 | 0.2255 |

**Supplementary Tables 5.** Two-way ANOVA of the effects of *B. cereus* on the population growth of *A. gossypii* at different initial densities

| Aphids density | Variables | F | P |
| --- | --- | --- | --- |
| 5 | species | 6.599 | 0.0172* |
|  | *A. gossypii* | 0.028 | 0.9721 |
|  | species**A. gossypii* | 5.826 | 0.0090** |
| 10 | species | 17.264 | 0.0004*** |
|  | *A. gossypii* | 4.784 | 0.0178* |
|  | species**A. gossypii* | 6.416 | 0.0059** |
| 15 | species | 62.854 | <0.0001*** |
|  | *A. gossypii* | 13.070 | 0.0002*** |
|  | species**A. gossypii* | 13.749 | <0.0001*** |

**Supplementary Tables 6.** Two-way ANOVA of the effects of addition with *B. cereus* on soil characteristics of *A. adenophora* fed by *A. gossypii* (15 nymphs)(Xia, 2023 #131)(Xia, 2023 #131)(Xia, 2023 #131)

|  | Variables | F | P |
| --- | --- | --- | --- |
| Moisture content | species | 0.507 | 0.4860 |
|  | *A. gossypii* | 0.181 | 0.8364 |
|  | species**A. gossypii* | 2.160 | 0.1459 |
| pH | species | 15.471 | 0.0006*** |
|  | *A. gossypii* | 21.327 | <0.0001*** |
|  | species**A. gossypii* | 4.678 | 0.0192* |
| NH_4_^+^-N | species | 956.382 | <0.0001*** |
|  | *A. gossypii* | 12.801 | 0.0003*** |
|  | species**A. gossypii* | 5.949 | 0.0104* |
| NO_3_^–^-N | species | 0.000 | 0.9981 |
|  | *A. gossypii* | 4.025 | 0.0359* |
|  | species**A. gossypii* | 2.183 | 0.1416 |
| AP | species | 5.323 | 0.0331* |
|  | *A. gossypii* | 1.372 | 0.2789 |
|  | species**A. gossypii* | 3.756 | 0.0433* |
| AK | species | 72.630 | <0.0001*** |
|  | *A. gossypii* | 234.749 | <0.0001*** |
|  | species**A. gossypii* | 62.067 | <0.0001*** |
